# Ultrasound-induced suppression of visually-evoked potentials: experience in nonhuman primates with a clinical transcranial MRI-guided focused ultrasound system

**DOI:** 10.1186/2050-5736-3-S1-O24

**Published:** 2015-06-30

**Authors:** Nathan McDannold, Costas Arvanitis, Natalia Vykhodtseva, Margaret Livingstone

**Affiliations:** 1 Brigham & Women’s Hospital/Harvard Medical School, Boston, MA, United States; 2 Harvard Medical School, Boston, MA, United States

## Background/introduction

Previous works by Fry *et al*. and others demonstrated that ultrasound exposures on the optic tract and/or the lateral geniculate nucleus (LGN) can temporarily block visually-evoked potentials in small animals. We attempted to repeat these experiments in a rhesus macaque using the ExAblate Neuro system (InSightec) operating at 650 kHz.

## Methods

The animal was deeply anesthetized using ketamine and dexdormitor. First we targeted the LGN using MR temperature imaging, and then we relocated the MRI table just outside the MRI. Visually-evoked potentials driven by a strobe light were recorded transcranially using electrodes placed over the visual cortex. Recordings were obtained before, during, and after sonications at different targets over a range of pulse parameters and acoustic power levels. In some cases the focal point was steered electronically to multiple targets during each sonication. In some sessions we tested whether disabling portions of the array to increase the length of the focal region could improve the outcome. We also confirmed the targeting at the end of the sessions by disrupting the blood-brain barrier using sonications with Optison microbubbles.

## Results and conclusions

We were able to reliably obtain strong visually-evoked potentials within the ExAblate system. Over seven sessions, a total of 133 sonications at 57 different targets on and around the LGN were evaluated. In no case were we able to significantly suppress the evoked potentials. In a few cases, large DC shifts that lasted several minutes were induced, perhaps indicative of cortical spreading depolarization. No tissue damage was evident in MRI, and the animal recovered and behaved normally after each session.

It is not clear why these experiments failed to suppress the evoked potentials as observed in other studies. We could not tell whether the targeting was bad, the focal exposure level was insufficient, incorrect pulsing parameters were used, or if other factors such as anesthesia were responsible. This work highlights the challenges inherent in this type of experiment.

